# Non-absorbable Barbed Sutures for Primary Fascial Closure in Laparoscopic Ventral Hernia Repair

**DOI:** 10.7759/cureus.22523

**Published:** 2022-02-23

**Authors:** Lisandro Montorfano, Samuel Szomstein, Roberto J Valera, Stephen J Bordes, Mauricio Sarmiento Cobos, Frederico P Quirante, Emanuele Lo Menzo, Raul J Rosenthal

**Affiliations:** 1 Surgery, Cleveland Clinic Florida, Weston, USA; 2 Bariatric and Minimally Invasive Surgery, Cleveland Clinic Florida, Weston, USA; 3 Surgery, Louisiana State University Health Sciences Center, New Orleans, USA

**Keywords:** mesh repair, laparoscopy, minimally invasive surgery, barbed sutures, ventral hernia repair

## Abstract

Purpose

The aim of this study is to describe the safety and effectiveness of laparoscopic ventral hernia repair with intraperitoneal fascial closure using a barbed suture prior to mesh placement.

Materials and methods

Patients who underwent laparoscopic ventral hernia repair were included in this retrospective review. Patients were divided into two groups. In the first group, primary fascial closure was performed with a 2-polypropylene non-absorbable unidirectional barbed suture followed by fixation of the intraperitoneal mesh. In the second group, the mesh was fixed intraperitoneally using tacks without closing the fascial defect.

Results

A total of 148 patients who underwent laparoscopic primary ventral hernia repair were included. A total of 72 (48.6%) patients were included in the barbed suture with mesh group and 76 (51.4%) patients in the mesh-only group. The mean fascial defect size was 25 cm^2^ in the first group and 64 cm^2 ^in the second group. The median suturing time for fascial closure was 15 minutes. The average surgery time was 98 minutes in the first group and 96 minutes in the second group. The mean follow-up period was 80 days for Group 1 and 135 days for Group 2. No hernia recurrence or mortality occurred in this study.

Conclusion

The barbed suture closure technique is a fast, safe, and effective technique for fascial closure during laparoscopic ventral hernia repair in combination with mesh placement. Further evidence to support these findings and longer follow-up periods are warranted to evaluate long-term outcomes.

## Introduction

Incisional hernias affect approximately 16-20% of patients undergoing laparotomy. Annually, billions of dollars are spent in the American healthcare system for incisional hernia repairs [1]. Ventral hernia repair has improved over the years. The laparoscopic approach, introduced in 1993, became popular due to superior outcomes when compared to open repairs [2-4]. Sauerland et al. reported that laparoscopic repair of abdominal wall hernias decreased wound infection rates and allowed for faster recovery [5].

Since the introduction of laparoscopic ventral hernia repair, many techniques have been described. Laparoscopic techniques are normally performed using mesh. The mesh supports the repair and reduces tension on the abdominal wall. Chowbey et al. proposed an onlay technique in which a mesh is placed extraperitoneal to minimize complications, such as fistula formation and adhesions [6]. The sublay technique places the mesh below the fascia and is associated with lower recurrence rates [7].

Regardless of mesh location, several authors suggested the closure of the fascial defect prior to mesh placement to reconstruct the abdominal wall. This restores the structural and functional continuity of the musculofascial system and provides stable and durable wound coverage [8,9]. However, suturing the anterior abdominal wall laparoscopically may be challenging for surgeons without laparoscopic training. Non-absorbable barbed sutures appear to be growing in popularity as a novel material for tissue fixation and approximation across different surgical specialties [10-12]. In a previous study, we first described the technique for laparoscopic ventral hernia repairs using non-absorbable barbed sutures to close the fascial defect intracorporeally [13]. In this study, we describe the safety and effectiveness of laparoscopic ventral hernia repair with intraperitoneal fascial closure using a non-absorbable barbed suture prior to mesh placement.

## Materials and methods

**Figure 1:**
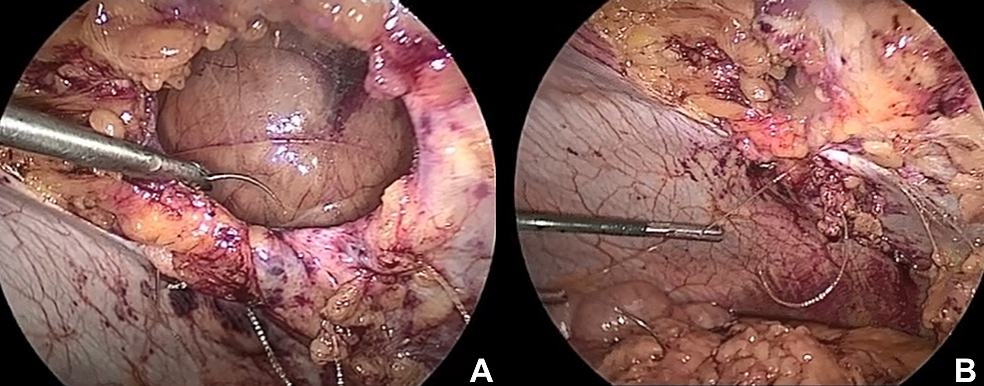
Laparoscopic view of fascial closure using a barbed suture. A: Fascial defect prior to closure. B: Fascial defect being closed with barbed sutures.

After Institutional Review Board (IRB) approval, a retrospective review was performed. Between 2013 and 2015, a total of 148 consecutive patients who underwent laparoscopic ventral hernia repair at our institution were analyzed. Cases that included the open conversion and primary non-incisional hernias were excluded. Only primary non-recurrent ventral hernias were included. None of the included patients exhibited rectus diastasis. We divided our population into two groups. In the first group (Group 1), the fascial defect was primarily closed with a barbed suture, and the mesh was subsequently fixed intraperitoneally (Figure 1). In the second group (Group 2), the mesh was tacked intraperitoneally and the fascial defect was not closed.

All procedures were performed by two board-certified surgeons trained in minimally invasive surgery. The non-absorbable barbed sutures used during this study were 2-polypropylene non-absorbable unidirectional Quill^TM^ sutures (Angiotech Pharmaceuticals, Inc., Vancouver, BC, Canada). The prosthesis used in all cases was the Parietex^TM^ composite ventral patch (Covidien, Mansfield, MA). Both procedures were performed in a standard fashion described in our prior publication [13]. Every patient received the same standardized postoperative care after surgery. All patients were discharged when they passed flatus and tolerated a clear liquid diet. Follow-up visits were scheduled two weeks postoperatively for all patients.

The data analyzed included age, gender, body mass index (BMI), defect area, mesh area, surgery time, suture time for the barbed suture with mesh group, length of stay, first and last follow-up after surgery, postoperative complaints within 30 days of surgery, and recurrence after the surgery. Continuous variables were described by their quartiles, and categorical variables were described by counts and percentages. Comparisons of categorical variables between groups were done using the Pearson chi-squared test for nominal variables and the proportional odds likelihood ratio test for ordinal variables. Comparisons of continuous variables between groups were performed using the Wilcoxon rank-sum test or the Kruskal-Wallis test. All analyses and summaries use complete cases only and were created using R software (R version 3.2.1 (2015-06-18), Vienna, Austria). A significance level of 5% was used for analysis.

## Results

A total of 148 patients who underwent laparoscopic ventral hernia repair were included in this study. A total of 72 (48.6%) patients underwent mesh fixation with primary fascial closure using barbed suture and 76 (51.4%) patients underwent mesh placement without closure of the fascial defect. Preoperative, intraoperative, and postoperative cohort characteristics can be found in Table 1. All patients were symptomatic preoperatively. Five patients were lost to follow-up (less than one month).

**Table 1:** Preoperative, intraoperative, and postoperative cohort characteristics.

	Group 1 (n = 72)	Group 2 (n = 76)	P-value
Sex (female)	32	50	0.009
Mean age (years)	58 (49-67)	59 (50-66)	0.8
Mean body mass index (kg/m^2^)	32 (28-37)	31 (27-35)	0.3
Patients with incarcerated hernias	34	14	0.001
Mean hernia defect (cm^2^)	25 (14-66)	64 (16-120)	0.06
Mean mesh area (cm^2^)	150 (81-225)	300 (225-500)	0.001
Mean barbed suture time (min)	15 (13-17)	Not applicable	Not applicable
Mean operative time (min)	98 (69-130)	96 (73-141)	0.4
Mean length of hospitalization (days)	1 (1-2)	2 (1-3)	0.001
Average time to follow-up (days)	11.05 (0-30, SD 5.4)	11.77 (1-22, SD 4.35)	0.07

We compared overall postoperative complaints and complications within 30 days of surgery (Table 2). Most were self-limited. There were two cases of major complications (hemorrhage and wound infection), which occurred in the mesh-only group.

**Table 2:** Comparison of postoperative complaints and complications between patients with and without barbed sutures.

	n	Mesh (n = 76)	Barbed suture with mesh (n = 71)	Test statistics
Nausea and vomiting	147	8% (6)	4% (3)	p = 0.4
Fevers	147	4% (3)	1% (1)	p = 0.3
Wound infection	147	5% (4)	0% (0)	p = 0.05
Incisional discomfort	147	63% (48)	51% (36)	p = 0.1
Bleeding	147	2% (1)	0% (0)	p = 0.4

The mean final follow-up was 80 days postoperatively (range 0-636 days, standard deviation = 137.91) for Group 1 and 135.05 days postoperatively (range 5-780 days, standard deviation = 226.78) for Group 2 (p < 0.001). No cases of hernia recurrence were documented in either of the groups. No mortality was documented in this study.

## Discussion

Intracorporal suturing can be challenging even for experienced surgeons. Although barbed sutures have been utilized more frequently over the last decade during laparoscopic surgery, there are limited data regarding their safety, efficacy, and complications in laparoscopic ventral hernia repair. Primary fascial closure restores normal anatomy and is suggested by some authors as an additional step necessary for a good hernia repair, thus reducing both short- and long-term complications [14,15]. In a recent systematic review published by Nguyen et al., it was shown that primary closure of the fascial defect improved abdominal wall function when compared to the standard bridging repair [16]. Placement of a mesh in an underlay fashion without fascial suturing of the defect may not provide the same support as a well-reconstructed abdominal wall, and the patient may still feel a protuberance at the surgical site when intra-abdominal pressure changes. As it is shown in our study, the size of the defect plays a significant role during closure. Not all defects were amenable to primary closure without tension secondary to their size. Some authors have proposed the component separation technique in the repair of complex abdominal wall hernias, advancing the abdominal muscles toward the midline and preserving the neurovascular bundles, which results in a dynamic, longstanding repair [17].

In regards to the surgical technique, the patient is placed in a supine position with the left arm tucked, the peritoneal cavity is entered at the level of the left upper quadrant (LUQ) by using a 5 mm (millimeter) optical trocar. After the establishment of pneumoperitoneum, a 5 mm trocar is placed in the left mid-quadrant lateral to the rectus muscle, and another 5 mm trocar is positioned in the left lower quadrant laterally. The LUQ trocar is then switched to a 12 mm trocar. If required, lysis of adhesions is performed by sharp or ultrasonic dissector and the hernia contents are reduced. The peritoneal sacs are excised/fenestrated to avoid seroma formation. Full-thickness fascial approximation of the ventral hernia defect is performed by a continuous suturing pattern with a 2-polypropylene non-absorbable unidirectional barbed suture. Each loop of barbed suture is accompanied by a decrease in pneumoperitoneum from 14 to 10 mmHg in reducing abdominal wall tension. The barbed suture is subsequently fixed in place by a single LAPRA-TY® suture clip (Ethicon Endo-Surgery, Cincinnati, OH) at the end of the suture. The polyester composite mesh size is selected to overlap 5 cm longitudinally and laterally of the primarily closed defect and then is inserted through the 12 mm trocar. In all cases, a single centering stitch was placed through the mesh and passed transfascially by a suture passer device. No transfascial sutures at the cardinal points of the mesh are required for mesh fixation. The mesh was circumferentially tacked at 1 cm intervals in a single crown pattern using the ProTack™ (Covidien, Mansfield, MA) in an underlay fashion. In cases where only mesh was used, mesh selection and placement followed the same protocol as described above [13].

Although we initially hypothesized that closing the defect would prolong operating room time, no difference was found between the two groups. Moreno-Egea et al. stated that surgical time increases as defect size increases [18]. It is important to note that the mean area of the ventral hernia defect was higher for the mesh-only group. However, the mean operation time for Group 1 (91 minutes) was shorter than the mean operation time reported by Venclauskas et al. (168.4 minutes) and by Israelsson et al. (102 minutes) using comparable techniques to close similar abdominal wall defects [19,20].

Our patients in the barbed suture with mesh group had a shorter duration of hospitalization and faster recovery time than those in the mesh-only group. The mean length of stay in the hospital in our study was one day (range one to two days) for the barbed suture with mesh group and two days (range one to three days) for the mesh-only group, which was relatively similar to our previous study in which we described the technique for the first time [13].

We had a follow-up rate of 98% within the first four weeks of surgery. The same post-surgical recommendations of rest and limited activity were given to all of our patients regardless of the surgical technique. Although the rates of nausea, vomiting, fever, chills, and incisional discomfort were higher in the mesh-only group, the differences observed were not statistically significant. Postoperative pain was assessed as a categorical variable and not using a visual analog scale [19]. In all cases, the pain was appropriately controlled with small doses of over-the-counter analgesics. There was no statistically significant difference in the rate of infection when the two techniques were compared.

Both procedures were successful in the early postoperative period with limited complications. Patients reported less abdominal discomfort when a barbed suture was used for primary closure. All of our patients were clinically symptomatic prior to surgery, secondary to chronic incarceration in many cases. It is reasonable to argue that increased reports of postsurgical discomfort in the mesh-only group may be due to a lack of integrity of the abdominal wall. As a result, the barbed suture may provide a balanced, knotless closure and a superior abdominal wall contour postoperatively.

In regard to long-term results, the mesh-only group had a longer postoperative follow-up period since 70% of the cases were performed in 2013 and 2014 while 58% of cases in the barbed suture group were performed in 2015. Venclauskas et al. reported a recurrence rate of up to 10.5% with a similar barbed suture closure technique, yet we found no hernia recurrences in either of the groups [20]. Primary fascial closure using laparoscopic ventral hernia repair with mesh reinforcement has been reported to decrease hernia recurrence rate [21]. Recurrent incisional hernias are likely related to the size of the hernia, which may make the use of mesh particularly important for incisional hernias larger than 4 cm [5,22]. However, additional studies with a longer follow-up period are warranted to draw more accurate conclusions.

Limitations include the retrospective nature of this study, the short duration of follow-up, and subjective measurement of postoperative pain. In addition, fascial defect sizes were not matched between groups.

## Conclusions

The barbed suture closure technique is a fast, safe, and effective technique for fascial closure during laparoscopic ventral hernia repair in combination with mesh placement. Further evidence to support these findings and longer follow-up periods are warranted to evaluate long-term outcomes.
